# Interpretable Multiscale Directed Temporal Graph Learning for MEG-Based Identification and Lateralization of Temporal Lobe Epilepsy

**DOI:** 10.3390/s26196185

**Published:** 2026-09-29

**Authors:** Yilin Jiang, He Wang, Jun Yan, Shuicai Wu, Ting Wu, Chunlan Yang

**Affiliations:** 1 College of Chemistry and Life Science, Beijing University of Technology, Beijing 100124, China; 2 Department of Radiology, Jiangsu Province Hospital of Chinese Medicine, Affiliated Hospital of Nanjing University of Chinese Medicine, Nanjing 210029, China

**Keywords:** TLE, MEG, directed brain network, GNN, lateralization, classification

## Abstract

**Objective**: To address the limitations of existing deep learning methods for brain networks in jointly modeling directional interregional connectivity, multiband information, and short-term dynamic features, this study proposes a multiscale directed temporal graph convolutional network (MSD-STGNN) for the three-class classification of healthy controls (HCs), patients with left temporal lobe epilepsy (lTLE), and patients with right temporal lobe epilepsy (rTLE). **Methods**: Resting-state magnetoencephalography (MEG) data were obtained from 43 subjects, including 14 HCs, 13 patients with lTLE, and 16 patients with rTLE. Based on 26 predefined default mode network (DMN)-related brain regions, directed effective connectivity networks were constructed in six frequency bands using the directed transfer function (DTF), and indices including information-flow strength, directional preference, and hemispheric asymmetry were used as node features. MSD-STGNN separately modeled incoming and outgoing connectivity information through directed graph convolution, fused frequency-band information using a hierarchical multiband attention mechanism with gated residual correction, and employed gated recurrent units (GRUs) to extract short-term dynamic features from consecutive brain-network slices. Model performance was evaluated using subject-level stratified fivefold cross-validation, with predictions from multiple temporal groups of each subject aggregated to obtain the final subject-level prediction. Frequency-band masking and node-level fusion-weight analyses were further performed to evaluate the model’s dependence on different frequency bands and information from the predefined brain regions. **Results**: In the subject-level fivefold cross-validation, MSD-STGNN achieved an accuracy of 0.836 ± 0.067, a macro-F1 of 0.830 ± 0.065, and a macro-AUC of 0.900 ± 0.055 using the one-vs-rest strategy, with the highest fivefold mean values across all evaluation metrics among the baseline models and ablation configurations investigated in this study. Post-training frequency-band masking showed that the model exhibited relatively high dependence on the low-gamma (30–80 Hz) and beta (13–30 Hz) bands. Node-level fusion-weight analysis showed that, within the 26 predefined DMN-related brain regions, orbitofrontal, cingulate, medial temporal, and parietal regions exhibited relatively high overall fusion weights across different frequency bands, although the exact top 5 regional rankings varied across folds. **Significance**: Within a unified framework, MSD-STGNN integrates directional connectivity, multiband information, and short-term dynamic features derived from MEG-based directed brain networks, providing **a** modeling approach with a certain degree of interpretability for the three-class classification of HCs, patients with lTLE, and patients with rTLE. The current findings are based on internal cross-validation of a small, single-center cohort; therefore, the classification performance and the observed frequency-band and brain-region attention patterns require further validation in larger, independent multicenter datasets.

## 1. Introduction

Epilepsy is a neurological syndrome caused by highly synchronized abnormal neuronal discharges in the brain due to various etiologies and is characterized by chronic and recurrent seizures [1]. It is one of the most common neurological disorders [2,3]. Temporal lobe epilepsy (TLE) is a common type of focal epilepsy in adults and is also an important form of drug-resistant epilepsy [4]. Studies have shown that abnormal brain activity in patients with TLE is not uniformly distributed across all frequency ranges but usually exhibits marked frequency-band specificity, with different frequency bands potentially corresponding to different neural activity patterns and abnormal propagation characteristics [5]. Magnetoencephalography (MEG) is a noninvasive neuroimaging technique capable of recording neural activity in the brain [6]. Compared with techniques such as electroencephalography and functional magnetic resonance imaging, MEG combines high temporal resolution with good spatial localization capability [7] and has unique advantages in epileptic focus localization and neural activity analysis [8,9,10]. Previous MEG studies have found that patients with TLE exhibit frequency-band-related power abnormalities and functional connectivity changes during the resting state [11,12,13,14].

In neuroscience, functional brain networks are a key concept for exploring the complex mechanisms underlying brain function [15]. An increasing number of studies have suggested that epilepsy is a network disorder [16] characterized by altered patterns of network interactions [17]. Graph neural networks (GNNs) have been widely used in brain network analysis [18,19,20]. By representing brain regions as nodes and interregional connection strengths as edges, GNNs can learn the topological structure of brain networks [21], making them well suited to brain network modeling.

At present, studies combining MEG with GNNs for epilepsy brain network analysis remain relatively limited, and existing deep learning methods for brain networks are mostly based on undirected functional or structural connectivity, with relatively little consideration of the directional characteristics of neural activity propagation between brain regions. In epilepsy, abnormal neural activity may involve not only changes in connection strength but also directional propagation between different brain regions [22]. The directed transfer function (DTF) is a frequency-domain effective connectivity analysis method based on multivariate autoregressive models [23], which can characterize directional information flow between brain regions at different frequencies and has been applied in epilepsy-related brain network studies [12,24,25,26]. On this basis, combining GNNs with multiband directed brain networks is expected to jointly utilize connectivity directionality and frequency-band-specific information, thereby providing a more comprehensive characterization of TLE-related brain network features.

Given the dynamic and continuously changing nature of brain activity, some studies have further combined GNNs with sequence modeling methods such as long short-term memory (LSTM) to jointly utilize graph-structured information and temporal dependency features [27,28]. From a broader biomedical engineering perspective, recent studies have increasingly integrated neurophysiological signal acquisition with deep learning, temporal modeling, and multimodal information fusion, providing new technical approaches for automated representation learning from complex physiological information and the development of intelligent biomedical systems [29,30].

Against this background, this study proposes a multiscale directed temporal graph convolutional network (MSD-STGNN). Based on multiband directed brain networks constructed using DTF, the model integrates directed graph convolution, multiband dynamic fusion, and temporal modeling to jointly characterize directional connectivity between brain regions, frequency-band-specific information, and short-term dynamic changes. The model is designed for the three-class classification of healthy controls (HCs), patients with left temporal lobe epilepsy (lTLE), and patients with right temporal lobe epilepsy (rTLE). In addition, interpretability analyses at the frequency-band and brain-region levels are performed to explore the discriminative information utilized by the model, providing a new modeling strategy for TLE classification and abnormal brain network feature analysis.

## 2. Materials and Methods

### 2.1. MEG Data

The data used in this study were obtained from Nanjing Brain Hospital, affiliated with Nanjing Medical University, China. The original dataset comprised 14 healthy controls (HCs), 13 patients with left temporal lobe epilepsy (lTLE), and 21 patients with right temporal lobe epilepsy (rTLE). To reduce class imbalance in the three-class classification task, all 14 HCs and 13 patients with lTLE were included, together with 16 patients from the rTLE group, resulting in a final cohort of 43 subjects. The 16 subjects in the rTLE group were selected according to the order of subject IDs, without reference to any classification results, model performance, or feature information. The clinical lateralization labels for lTLE and rTLE were determined by Nanjing Brain Hospital before the data were provided, based on clinical evaluation incorporating electroencephalography (EEG), MEG findings, and clinical manifestations; the lateralization results of some patients were further confirmed intraoperatively. This study did not participate in the original clinical lateralization assessment, nor were the original group labels modified according to subsequent MEG-derived network features, model predictions, or any other analysis results. All patients with TLE were diagnosed with drug-resistant epilepsy, and structural magnetic resonance imaging (sMRI) showed no evident abnormalities in most cases. The HCs had no history of psychiatric disorders or sMRI abnormalities, and all subjects were right-handed. No significant differences in age or sex were observed among the three groups (age: one-way ANOVA, F = 0.354, *p* = 0.704; sex distribution: χ^2^ = 2.693, *p* = 0.260). The demographic and clinical characteristics of the subjects included in this study are presented in Table 1.

MEG data were acquired using a 275-channel whole-head MEG system (VSM MedTech Systems Inc., Coquitlam, BC, Canada) at an original sampling frequency of 4 kHz. During acquisition, a synthetic third-order gradient technique was used to suppress environmental noise. Resting-state MEG recordings were obtained while the subjects were awake, with their eyes closed, and in the supine position. Head position was recorded at both the beginning and end of the experiment, and data with head displacement greater than 5 mm were excluded. A total of 20 resting-state MEG recordings, each lasting approximately 120 s, were acquired from each subject. After inspection by trained personnel, one 120 s interictal recording without obvious epileptiform activity was selected for subsequent analysis. sMRI data were acquired using a GE Signa NV/i 1.5 T magnetic resonance imaging scanner (GE Healthcare, Milwaukee, WI, USA). Written informed consent was obtained from all subjects before data acquisition.

### 2.2. Data Preprocessing

The raw MEG signals were preprocessed by filtering and independent component analysis (ICA) to obtain clean MEG signals. First, a 0.5 Hz high-pass filter was applied to remove baseline drift, followed by a 50 Hz notch filter to suppress power-line interference. ICA was then used to remove artifacts such as electromyographic, electrooculographic, and electrocardiographic artifacts from the MEG signals [31]. After artifact removal, the MEG data were resampled from the original 4 kHz to 500 Hz using the MNE-Python 0.23.0 toolbox. This downsampling procedure included anti-aliasing processing to suppress frequency components above the Nyquist frequency after resampling and reduce the effects of spectral aliasing. The resampled MEG data were subsequently segmented into 2 s epochs for subsequent source reconstruction. The entire MEG preprocessing pipeline was implemented using the MNE-Python toolbox.

**Figure 1:**
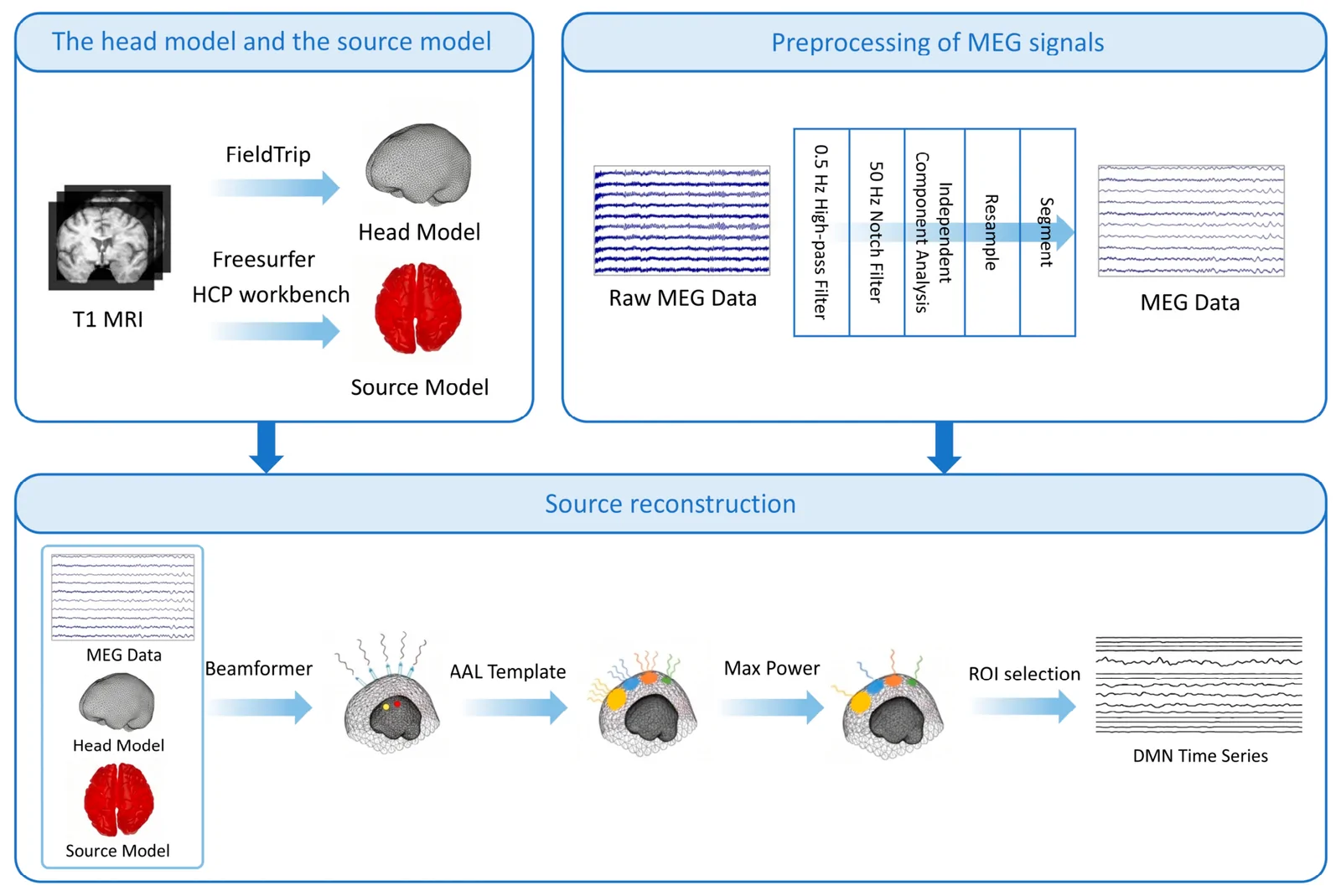
Data Preprocessing Flow.

To reconstruct the MEG signals into brain-region time series, source reconstruction was performed as a prerequisite for the subsequent construction of directed brain networks and node features using brain regions as nodes. Source reconstruction first required the construction of a head model and a source model. The head model was constructed using the FieldTrip 2019 toolbox to describe head geometry and tissue conductivity, whereas the source model was constructed using FreeSurfer 6.0 and HCP Workbench 1.5.0 to describe the positions and orientations of equivalent current dipoles. Source reconstruction was subsequently performed using the beamforming method [32], and the whole brain was parcellated into 116 regions using the Anatomical Automatic Labeling (AAL) template [33]. The signal with the maximum power in each brain region was selected to represent the regional time series. The 2 s regional time-series segments were then concatenated in their original temporal order, yielding continuous time series comprising 116 brain regions and 60,000 time points for each subject. The data preprocessing workflow is shown in Figure 1.

Previous studies have shown that the default mode network (DMN) plays an important role in the network pathophysiological mechanisms of temporal lobe epilepsy, and abnormal DMN connectivity may be associated with seizure propagation, altered consciousness, and cognitive impairment in patients with TLE [22,34,35]. Therefore, based on previous studies, 26 DMN-related brain regions were predefined as regions of interest (ROIs) in this study [36,37,38], as listed in Table 2. Finally, a 26 × 60,000 ROI time-series matrix was obtained for each subject and used for the subsequent construction of directed connectivity matrices and node features.

### 2.3. Construction of Adjacency and Node Feature Matrices

#### 2.3.1. Adjacency Matrix

This study employed the directed transfer function (DTF) to characterize directional information flow and effective connectivity strength among the time series of different brain regions [23,39]. DTF is based on a multivariate autoregressive (MVAR) model, which can be expressed as follows:

(1) $$X(t)=\sum_{k=1}^{p}\;A(k)*X(t-k)+E(t).$$

where X(t) represents an N-dimensional brain-region time series, A(k) denotes the *k*-th-order MVAR coefficient matrix, E(t) is the residual term, and *p* is the order of the MVAR model. In this study, *p* was uniformly set to 5, following the processing scheme previously adopted by our research group for the same MEG dataset and DTF analysis framework, and was kept consistent across all subjects, frequency bands, and temporal slices [12]. Sensitivity analyses for neighboring MVAR orders, slice-wise stability assessment, and residual diagnostics are provided in Supplementary Notes S1 and S2. DTF calculation was implemented using the eConnectome toolbox, with the MVAR model parameters estimated using the ARfit package [40]. Transforming Equation (1) into the frequency domain yields the frequency-domain coefficient matrix:

(2) $$A\left(f\right)=I-\overset{p}{\underset{k=1}{\sum }}\;A\left(k\right)e^{-j2\pi fk/f_{s}}.$$

where $f_{s}$ denotes the sampling frequency. The frequency-domain MVAR model and its transfer relationship can be expressed as follows:

(3) $$A\left(f\right)X\left(f\right)=E\left(f\right)\;\Rightarrow \;X\left(f\right)=A^{-1}\left(f\right)E\left(f\right)=H\left(f\right)E\left(f\right),D_{ij}\left(f\right)={\left|H_{ij}\left(f\right)\right|}^{2}.$$

where $H\left(f\right)$ = $A^{-1}\left(f\right)$ denotes the frequency-domain transfer matrix. The element $H_{ij}\left(f\right)$ represents directional information transfer from brain region *j* to brain region *i*. $D_{ij}\left(f\right)$ represents the connectivity strength of the non-normalized DTF. Throughout this study, the matrix element $D_{ij}$ consistently denotes a directed connection from node *j* to node *i*.

The DMN time series obtained in Section 2.2 were segmented into 40 non-overlapping temporal slices for each subject. Each slice contained 1500 time points, corresponding to 3 s at a sampling rate of 500 Hz. For each MEG slice, normalized and non-normalized DTF measures were calculated for the following six frequency bands: delta (1–4 Hz), theta (4–8 Hz), alpha (8–13 Hz), beta (13–30 Hz), low gamma (30–80 Hz), and high gamma (80–250 Hz) [7,12,41,42,43]. Within each frequency band, DTF connectivity was calculated at each frequency point and then averaged along the frequency dimension to obtain the corresponding band-specific DTF matrix. The non-normalized DTF matrix represents the absolute strength of interregional connectivity and was used to construct the node feature matrix. The normalized DTF matrix represents the relative strength of interregional connectivity and was used as the adjacency matrix input to the deep learning model. It should be noted that, at the resampled sampling rate of 500 Hz, 250 Hz corresponds to the Nyquist frequency. Therefore, in the DTF implementation used in this study, 250 Hz represents the Nyquist boundary of the discrete-time–frequency-response range rather than a frequency component above the Nyquist frequency. The high-gamma DTF representation was obtained by averaging the connectivity estimates across the entire 80–250 Hz range, and no independent neurophysiological interpretation was assigned to the 250 Hz boundary point itself. Log-scaled max normalization was applied as follows:

(4) $$D_{ij}^{log}\left(f\right)=\log\left[D_{ij}\left(f\right)+1\right],{\tilde{D}}_{ij}\left(f\right)=\frac{D_{ij}^{log}\left(f\right)}{\underset{m,n}{max}\;D_{mn}^{log}\left(f\right)},W_{ij}^{\left(b\right)}=\frac{1}{N_{b}}\underset{f\in F_{b}}{\sum }\;{\tilde{D}}_{ij}\left(f\right).$$

where $F_{b}$ denotes the set of frequency points contained in the *b*-th frequency band, and $N_{b}$ denotes the corresponding number of frequency points. After normalization, the diagonal elements were set to zero. This procedure reduces the influence of relatively large connectivity values on the numerical distribution through logarithmic compression and scales the DTF matrices at different frequency points to a common range, making them more suitable as inputs to the GNN [44].

#### 2.3.2. Node Features

Node features were calculated based on the non-normalized DTF matrix of each frequency band to characterize the information-flow strength and directional properties of nodes in the brain network. The following features were calculated:

Node in-strength [45]. This feature represents the total information flow received by a node from other nodes. It is defined as follows:

(5) $$S_{i}^{in}=\sum_{j=1}^{n}\;d_{ij}.$$

Node out-strength [45]. This feature represents the information flow transmitted from a node to other nodes. It is defined as follows:

(6) $$S_{i}^{out}=\sum_{j=1}^{n}\;d_{ji}.$$

Node directional preference [46]. This feature measures whether a node is predominantly input- or output-driven. Here, the input preference of a node is defined. In particular, $D_{i}>0\;$ indicates that the node receives additional information and is therefore likely to be driven by other nodes, whereas $D_{i}<0\;$ indicates that the node sends additional information and is therefore likely to actively drive other nodes. It is defined as follows:

(7) $$D_{i}=\frac{S_{i}^{in}-S_{i}^{out}}{S_{i}^{in}+S_{i}^{out}+\varepsilon }.$$

where ε is a small constant used to prevent division by zero.

Node input power. This feature emphasizes the contribution of strong information pathways. In this feature construction, strong connections are amplified, whereas the influence of weak connections is reduced. It is defined as follows:

(8) $$P_{i}=\sum_{j=1}^{N}\;A_{ij}^{2}.$$

Differential features based on corresponding left–right brain regions were further introduced [47]. Assuming that node $i^{\prime }$ is the contralateral node corresponding to node *i*, the following features were defined:

Asymmetry of node in-strength relative to the contralateral node (Asym In-strength). This feature measures the imbalance in information reception between corresponding left and right brain regions. A positive value indicates that the in-strength of brain region *i* is greater than that of its contralateral paired region.

(9) $$\Delta S_{i}^{in}=\frac{S_{i}^{in}-S_{i^{\prime }}^{in}}{S_{i}^{in}+S_{i^{\prime }}^{in}+\epsilon }.$$

Asymmetry of node out-strength relative to the contralateral node (Asym Out-strength). This feature measures the difference in information output between corresponding left and right brain regions.

(10) $$\Delta S_{i}^{out}=\frac{S_{i}^{out}-S_{i^{\prime }}^{out}}{S_{i}^{out}+S_{i^{\prime }}^{out}+\epsilon }.$$

Asymmetry of node directional preference relative to the contralateral node (Asym Directionality). This feature measures the difference in information-flow direction between corresponding left and right brain regions, indicating whether one side tends to receive information while the other side tends to send information.

(11) $$\Delta D_{i}=\frac{D_{i}-D_{i^{\prime }}}{|D_{i}|+|D_{i^{\prime }}|+\epsilon }.$$

Asymmetry of node input power relative to the contralateral node (Asym Power). This feature measures lateralized difference in information flow dominated by strong connections.

(12) $$\Delta P_{i}=\frac{P_{i}-P_{i^{\prime }}}{P_{i}+P_{i^{\prime }}+\epsilon }.$$

Finally, a node feature matrix $X\in R^{26\times 8}$ was obtained. The 1st, 2nd, and 4th columns of the node feature matrix, namely, $S_{i}^{in}$, $S_{i}^{out}$, and $P_{i}$, were transformed as follows:

(13) $$x^{\prime }=log\left(1+max(x,0)+\varepsilon \right).$$

where ε is a small constant used to prevent division by zero. For possible negative values, the values were first clipped to 0 before transformation. This processing reduces the influence of extreme values on subsequent deep learning while preserving the relative scale patterns of the features.

### 2.4. Models

MSD-STGNN was proposed for modeling and classifying multiband brain networks. This model consists of three components: directed graph convolution, multiscale cross-band modeling, and temporal modeling.

The directed graph convolutional layer is the core component of the intraband spatial modeling module. For each time slice and each frequency-band-specific adjacency matrix, the model first uses the directed graph convolutional layer to extract spatial representations from node features and directed adjacency matrices. Traditional GCNs typically construct normalized adjacency matrices based on undirected graphs and perform feature propagation through a single pathway, thus failing to distinguish between information inflow and outflow. According to the DTF direction convention defined above, the original adjacency matrix A is used to aggregate information from incoming neighbors, whereas its transpose is used to aggregate information from outgoing neighbors. This study therefore decomposes directed graph convolution into three components: an incoming-edge branch, an outgoing-edge branch, and a self-feature pathway:

(14) $$H=\sigma \left(W_{in}(A^{in}X)+W_{out}(A^{out}X)+W_{s}X\right).$$

where *σ* denotes the nonlinear activation function, implemented using ReLU. $W_{in}(A^{in}X)$ is used to model the information received by a node from its incoming neighbors, emphasizing the influence of external inputs on the node state. $W_{out}(A^{out}X)$ is used to model the propagation relationship between a node and its outgoing neighbors, emphasizing the topological role of the node as an information source. $W_{s}X$ is used to preserve the original node attributes, preventing oversmoothing after multiple rounds of propagation while enhancing local feature representation. For directed brain networks constructed using DTF, this design explicitly preserves connectivity direction during message passing and separately models the incoming- and outgoing-neighbor relationships of each node [48].

To fully model hierarchical dependencies among different frequency bands, this study designed a node-level, dynamic, frequency-band attention fusion module. The six frequency bands were divided into the following two groups according to their frequency ranges to reduce the difficulty of direct fusion caused by excessive semantic differences across frequency bands: a low-frequency group, including 1–4 Hz, 4–8 Hz, and 8–13 Hz, and a medium-to-high-frequency group, including 13–30 Hz, 30–80 Hz, and 80–250 Hz. Intragroup self-attention modeling and intragroup dynamic weight generation were first performed within each frequency-band group. A multihead self-attention mechanism was applied within each group to capture the correlations and dependencies among different frequency bands. Residual connections and layer normalization were introduced after the intragroup attention operation to improve training stability [49]:

(15) $$Z_{g}=MHA(H_{g},H_{g},H_{g}).$$

(16) $${\hat{H}}_{g}=LayerNorm(Z_{g}+H_{g}).$$

After intragroup attention modeling, the features of the three frequency bands were not directly averaged. Instead, a lightweight multilayer perceptron was further used to generate dynamic intragroup weights [50,51]:

(17) $$s_{g,k}={MLP}_{intra}({\tilde{h}}_{g,k}).$$

where $MLP_{intra}$ consists of two linear mappings, ReLU activation, and dropout. A softmax function with a temperature parameter τ was then used to convert the scores into normalized weights [52]:

(18) $$\alpha_{g,k}=\frac{exp(s_{g,k}/\tau )}{\sum_{j=1}^{3}\;exp(s_{g,j}/\tau )}.$$

where τ denotes the temperature coefficient. When τ is large, the weight distribution becomes smoother; when τ is small, the model tends to emphasize a small number of dominant frequency bands. This parameter was used to regulate the competition strength among frequency bands within each group.

Finally, the intragroup representation was obtained by weighted summation. The intragroup attention module models the relationships among frequency bands, and the dynamic weight generator further determines which frequency band is important for the current node.

(19) $$r_{g}=\sum_{k=1}^{3}\;\alpha_{g,k}h_{g,k}.$$

After the low-frequency group representation $r_{L}$ and medium-to-high-frequency group representation $r_{H}$ were obtained, interactions between different frequency hierarchies were further modeled at the intergroup level. A multihead self-attention mechanism was used to capture intergroup dependencies, with residual connections and layer normalization retained. This step allows the model to establish associations between the two hierarchical levels of “low-frequency global modulation” and “medium-to-high-frequency local rapid activity,” thereby capturing complementary information across frequency levels. Intergroup dynamic fusion was then performed. Similar to intragroup fusion, intergroup weights were further calculated for the two frequency groups. In this way, the model can adaptively select key frequency bands within each group and dynamically determine whether the current node relies on low-frequency information or medium-to-high-frequency information at the intergroup level.

Hierarchical attention fusion can improve representational flexibility. However, relying entirely on attention weights may lead to excessive bias, thereby affecting model performance. Therefore, this study designed a gated residual correction mechanism [53]. First, the two group representations were averaged to obtain a baseline representation:

(20) $$h^{base}=\frac{1}{2}(r_{L}+r_{H}).$$

A scalar gate value was then generated through a gating network:

(21) $$g=\sigma ({MLP}_{gate}(h^{base})).$$

where σ(·) denotes the sigmoid function and g∈(0,1). The final fused representation is defined as follows:

(22) $$h^{fused}=h^{base}+g\cdot (h^{attn}-h^{base}).$$

This design is equivalent to adaptive interpolation between the “mean-fusion representation” and the “attention-fusion representation.” When the gate value g is small, the model tends to conservatively preserve the baseline information; when g is large, the model relies more on the dynamic fusion result learned by the attention mechanism.

To further characterize the dynamic evolution of brain networks across consecutive time slices, after obtaining the unified spatial representation of nodes at each time slice, gated recurrent units (GRUs) were used to model the temporal segments and extract global dynamic features. GRUs can alleviate the vanishing-gradient problem in traditional recurrent neural networks through gating mechanisms and effectively capture short- and medium-range sequential dependencies, making them suitable for modeling dynamic changes in short time series [54,55].

Since the importance of different brain-region nodes within the same time slice is not identical, directly averaging all nodes may weaken the discriminative contribution of key brain regions. Therefore, this study introduced a node attention pooling mechanism at each time step to assign adaptive weights to nodes. First, a two-layer perceptron was used to calculate an attention score for each node:

(23) $$e_{t,i}=W_{2}\left(Dropout(tanh(W_{1}h_{t,i}))\right).$$

where $W_{1}$ and $W_{2}$ are learnable parameters, and output $e_{t,i}\;$ denotes the attention score of node i at time slice t. Softmax normalization was subsequently applied to the scores of all nodes to obtain the node attention weights:

(24) $$\alpha_{t,i}=\frac{exp(e_{t,i})}{\sum_{j=1}^{N}\;exp(e_{t,j})},\sum_{i=1}^{N}\;\alpha_{t,i}=1.$$

A time-slice-level representation was then obtained by weighted summation:

(25) $$z_{t}=\sum_{i=1}^{N}\;\alpha_{t,i}h_{t,i}.$$

Finally, the original node-level feature sequence was compressed into a time-slice-level feature sequence while preserving the differences in the contributions of key brain regions at each time step.

**Figure 2:**
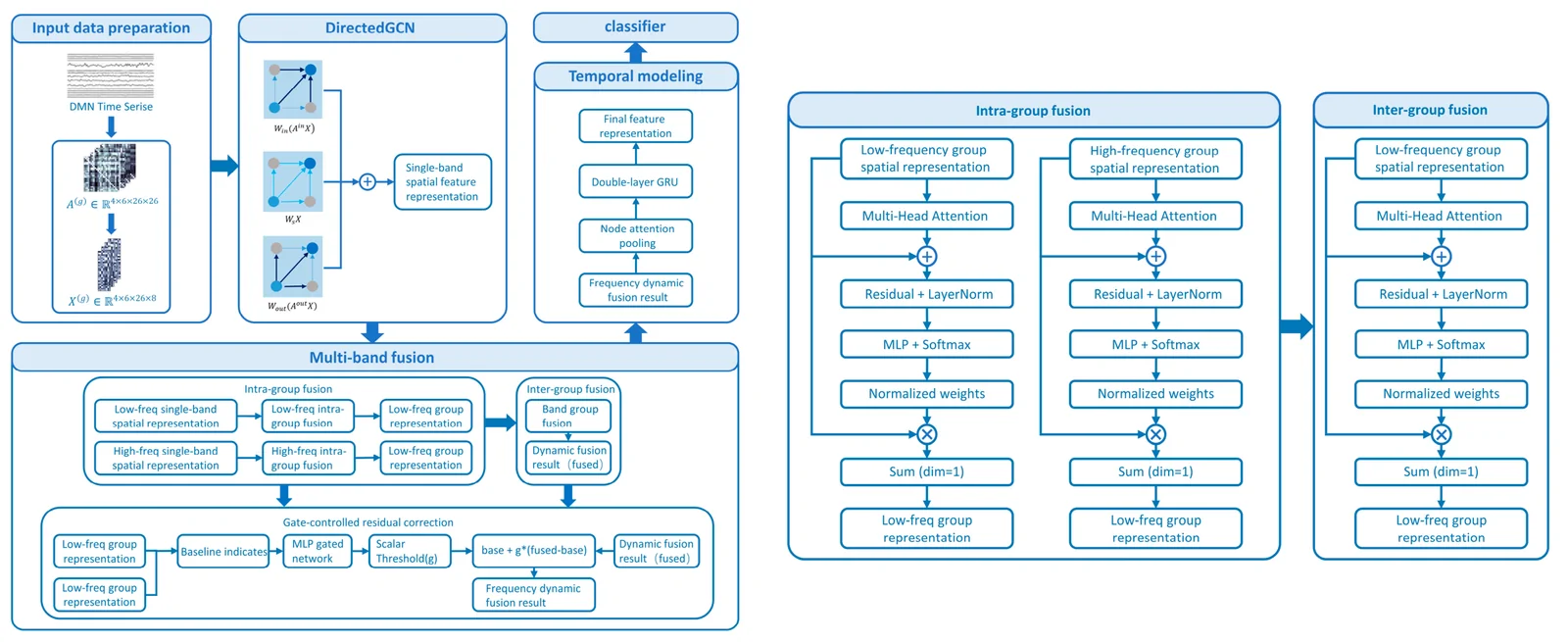
Overall Model Workflow. In the DGCN module workflow, the arrows in the node diagrams are purely schematic and are used only to illustrate directed information propagation between nodes; they do not represent specific edge weights or exact connectivity patterns. Colors are used only for visual distinction and do not encode additional quantitative information. In the detailed frequency-band fusion workflow, the “+” and “×” symbols denote addition and weighting operations, respectively.

In this study, a two-layer GRU was designed, and its recursive form can be expressed as follows:

(26) $$h_{t}^{\left(l\right)}={GRU}^{\left(l\right)}(h_{t-1}^{\left(l\right)},x_{t}^{\left(l\right)}).$$

where l denotes the GRU layer index. The input to the first layer is $x_{t}^{\left(1\right)}=z_{t}$, whereas the input to higher layers is the hidden state of the previous layer. Dropout was introduced between layers to enhance the generalization ability of the model. The overall model workflow is shown in Figure 2.

### 2.5. Training Procedure

The DTF effective connectivity matrices of the six frequency bands described in Section 2.3 and their corresponding node feature matrices were organized into temporal groups, with every four consecutive slices forming one group. Each subject was sequentially divided into 10 temporal groups. Accordingly, the temporal-group samples composed of adjacency matrices and node feature matrices had the following dimensions:

(27) $$A^{\left(g\right)}\in R^{4\times 6\times 26\times 26},\;\;X^{\left(g\right)}\in R^{4\times 6\times 26\times 8}.$$

The first dimension represents four consecutive slices in chronological order, the second dimension corresponds to the six frequency bands, and the third and fourth dimensions represent the nodes and their features, respectively. This organization serves two purposes. First, four consecutive slices form a short-term dynamic brain-network sequence, providing the input for temporal modeling. Second, without increasing the number of independent subjects, each subject is organized into 10 temporal groups, thereby increasing the number of within-subject temporal observations available to the model. These temporal groups are treated as repeated observations within each subject rather than as independent subject samples. To prevent data leakage, all temporal groups from the same subject were always assigned to the same data partition, and subject-level stratified fivefold cross-validation was employed. In each fold, model parameters were optimized using only the training set. A subject-level within-fold validation set comprising 20% of the training subjects was further separated from the training set, and the optimal model checkpoint was selected according to the macro-F1 score on the validation set. The selected checkpoint was then evaluated on the independent test set of that fold. The test set was not used for early stopping, hyperparameter tuning, or model checkpoint selection. During training, class-weighted cross-entropy loss was employed, with class weights determined according to the sample frequencies of the respective classes in the current training set.

Model performance was evaluated using fivefold cross-validation. The evaluation metrics included accuracy, precision, recall, specificity, F1-score, and area under the receiver operating characteristic curve (AUC). Five comparison and ablation experiments were further conducted to evaluate the effects of the directed effective-connectivity adjacency matrix, the multiscale frequency-band fusion strategy, the gated correction strategy, and the temporal modeling module on model performance.

In addition to temporal-group-level evaluation, subject-level prediction aggregation was further performed. For each subject, the predicted classes of the 10 temporal groups were aggregated by majority voting, and the class receiving the largest number of votes was taken as the final prediction [56]. Because all temporal groups were constructed using the same procedure and had the same duration, equal voting weights were assigned to them to reflect the overall prediction consistency across multiple temporal observations from the same subject. In the event of a tie for the highest number of votes, the final predicted class was determined according to the mean Softmax probability of the tied classes across the 10 temporal groups. Each test subject therefore generated only one final subject-level prediction in the corresponding cross-validation fold. Because temporal groups are not mutually independent clinical samples, temporal-group-level results were used only as an auxiliary evaluation, whereas subject-level results were regarded as the primary performance evaluation. Model parameters are provided in the Appendix A.

Subject-level accuracy and the confusion matrix were calculated from the final classes obtained by majority voting. Precision, recall, specificity, and F1-score were calculated using macro-averaging across the three classes. For ROC and AUC analysis, the mean Softmax probabilities of the 10 temporal groups for each class were used as continuous prediction scores, and macro-AUC was calculated using a one-vs-rest (OvR) strategy. Each metric was calculated separately for the five test folds, and the mean and standard deviation across the five folds were reported.

Model interpretability was mainly evaluated through frequency-band importance analysis and node-level fusion-weight analysis. Frequency-band importance was assessed using a post-training masking strategy. During testing, the adjacency matrices and node features corresponding to the target frequency band were set to zero without retraining the model, and the decrease in accuracy before and after masking was used to indicate the model’s dependence on that frequency band. Node-level fusion weights were used to analyze the brain regions receiving greater attention from the model within each frequency band. For each subject, node weights were first averaged across the four slices within each temporal group and then across the 10 temporal groups to obtain subject-level node-by-frequency-band weights. The mean and standard deviation were then calculated across the 43 subjects pooled from the test sets of the five folds, and the number of folds in which each brain region appeared among the top 5 regions of the corresponding frequency band was also recorded. Pairwise Jaccard indices were further calculated among the five top 5 brain-region sets for each frequency band, and the mean of the 10 pairwise comparisons was used to assess cross-fold stability. In addition, a random reference level was obtained by randomly generating top 5 sets from the 26 candidate ROIs and repeating the simulation 10,000 times. The observed cross-fold overlap was compared with the random reference level for descriptive purposes.

## 3. Results

**Figure 3:**
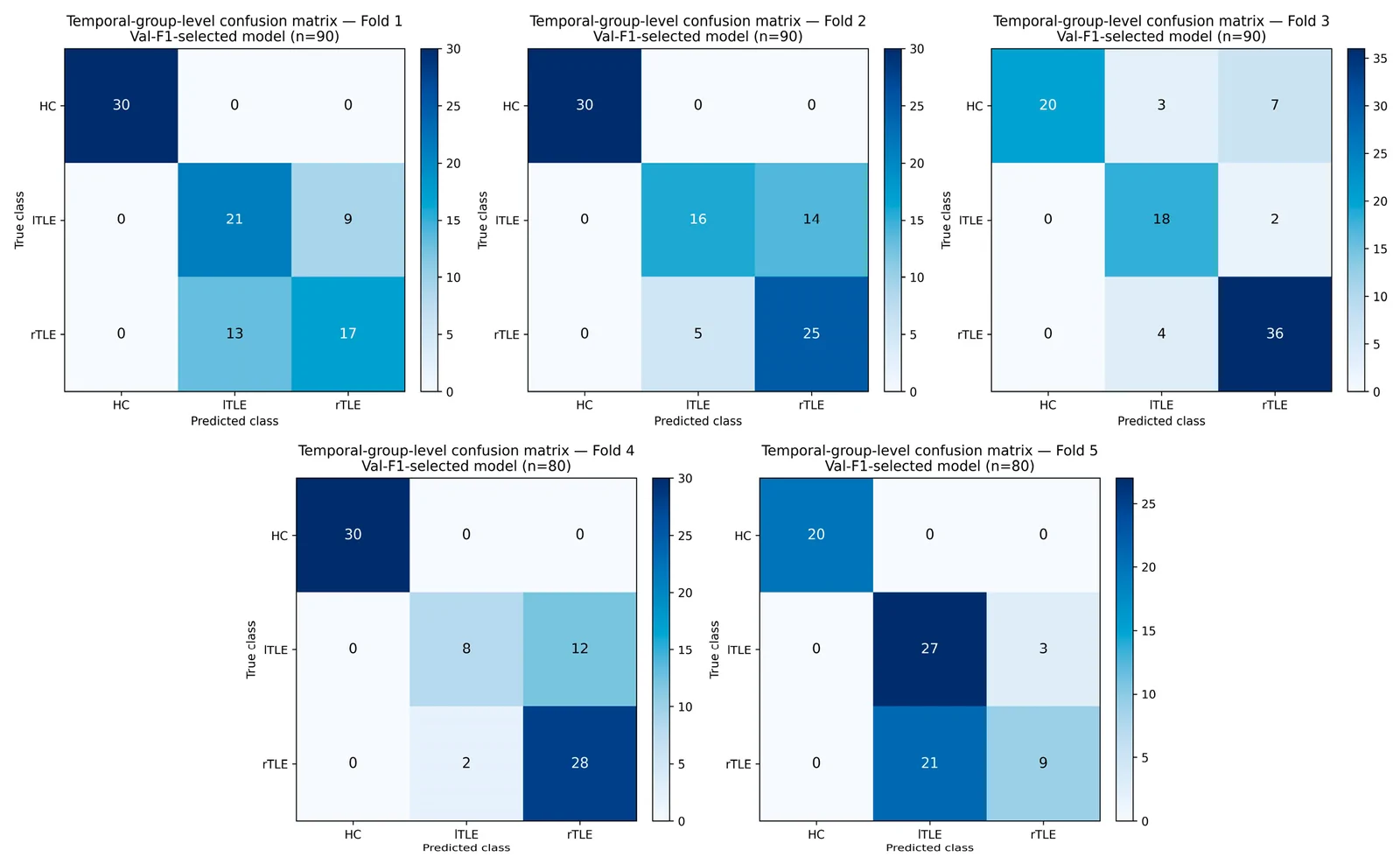
Fivefold temporal-group-level confusion matrices of the model selected by validation macro-F1, with classes HC, lTLE, and rTLE.

**Figure 4:**
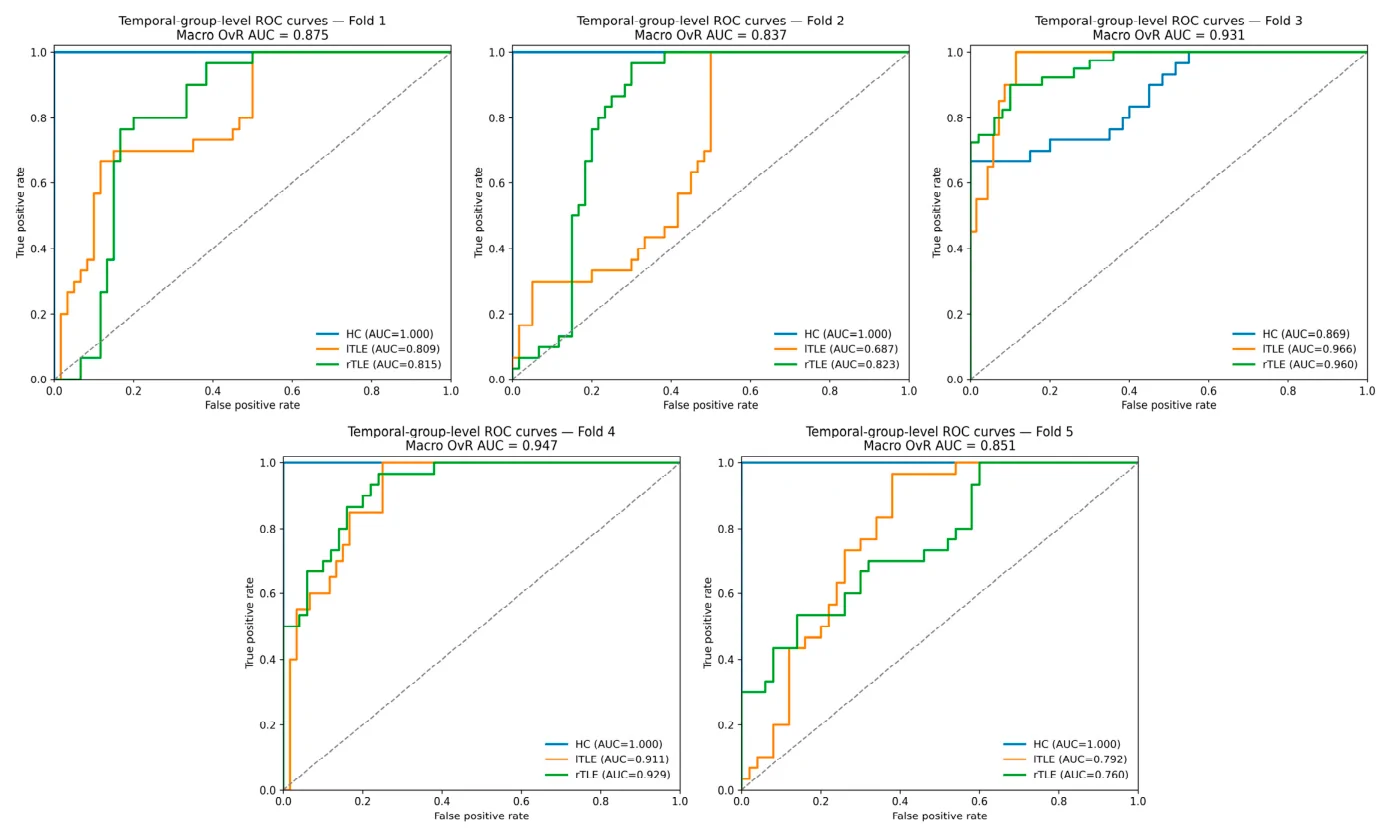
Fivefold temporal-group-level OvR ROC curves of the model selected by validation macro-F1.

Overall, in the fivefold cross-validation using subjects as the primary evaluation unit, MSD-STGNN achieved the highest mean values across all evaluation metrics among all methods examined in this study (Table 3). In the auxiliary temporal-group-level evaluation, the proposed MSD-STGNN also achieved the highest mean values across all evaluation metrics. Table 4 presents the class-specific evaluation metrics of the main model across the five folds. Figure 3 and Figure 4 show the confusion matrices and ROC curves for each fold of the temporal-group-level fivefold cross-validation, respectively. These results indicate that the model can distinguish HCs from patients with TLE relatively well, although some confusion remains in differentiating patients with lTLE from those with rTLE.

In the PLI baseline, an undirected adjacency matrix was constructed using PLI, while the subsequent model architecture was kept largely unchanged. The model without frequency-band fusion directly used a connectivity matrix constructed from the full frequency range of 1–250 Hz. The basic frequency-band fusion model retained all six frequency bands but used only a set of global learnable weights for frequency-band weighting. The model without gated correction retained the node-level dynamic frequency-band attention fusion mechanism but removed the gated residual correction module. The model without temporal modeling removed the temporal module and performed classification using only the features extracted by directed graph convolution and multiband fusion. The mean performance of all these comparison and ablation configurations was lower than that of the complete MSD-STGNN at both the subject level and the temporal-group level. Given that the fivefold cross-validation comprised only five folds and that the same subjects were repeatedly used as training data across different folds, no formal statistical significance inference was performed on the basis of the fivefold-level results. Accordingly, differences between models were primarily treated as descriptive comparisons.

**Figure 5:**
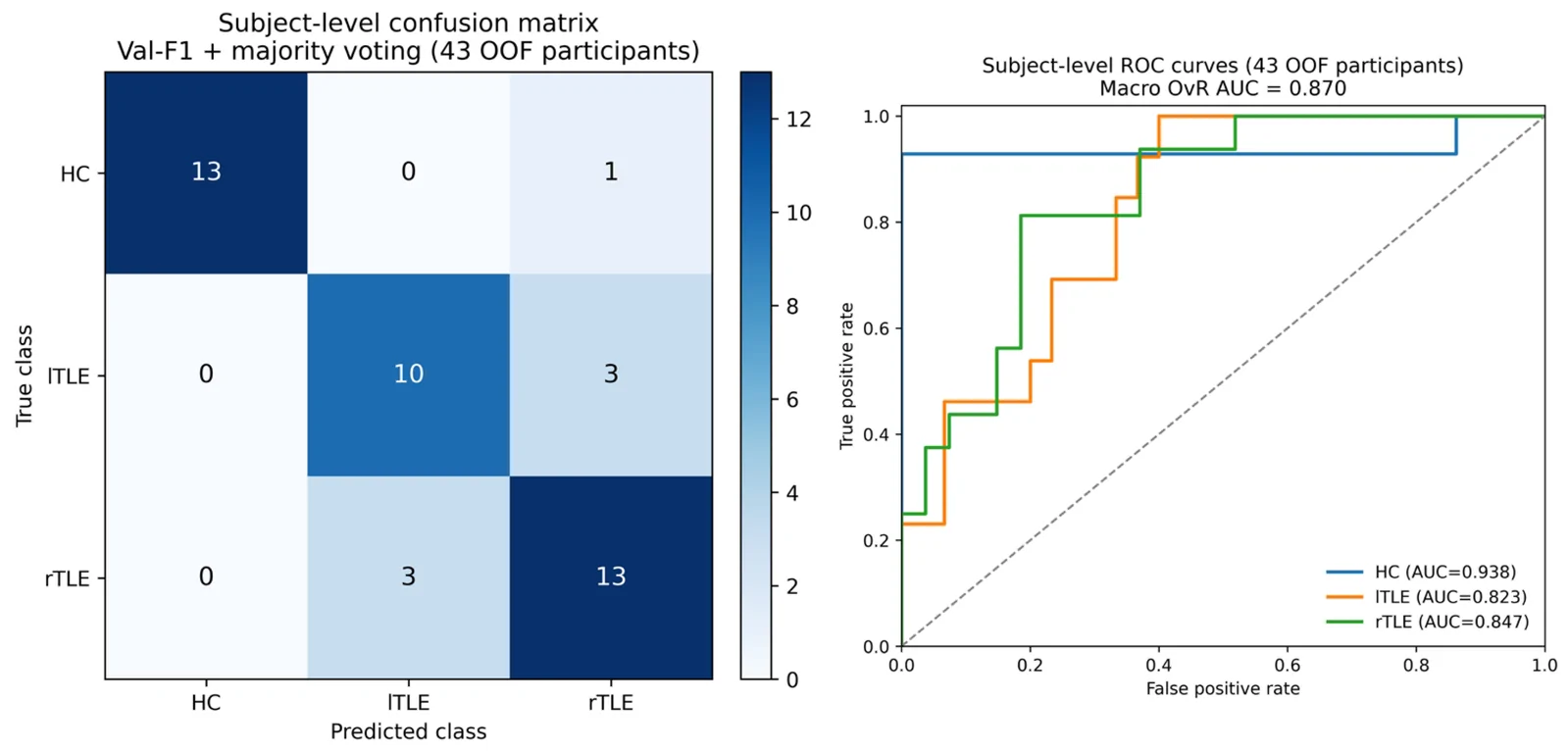
Pooled subject-level confusion matrix and ROC curves for the 43 OOF subjects. Final classes were determined by majority voting across the 10 temporal groups, and ROC curves were calculated from the mean Softmax probabilities across the 10 temporal groups.

The subject-level out-of-fold (OOF) predictions from the test folds of the fivefold cross-validation were pooled to provide a more intuitive overview of the model’s overall classification performance across all subjects. Each subject underwent independent testing only once, in the single cross-validation fold in which that subject was assigned to the test set. Therefore, the pooled OOF results included all 43 subjects without repeated testing. A pooled confusion matrix was constructed based on the final subject-level classes obtained by majority voting, while a pooled ROC curve was generated using the mean Softmax probabilities across the 10 temporal groups for each subject, as shown in Table 5 and Table 6 and Figure 5. The pooled results further showed that the model was able to distinguish HCs from subjects with TLE relatively well, whereas misclassifications were mainly concentrated between lTLE and rTLE.

**Figure 6:**
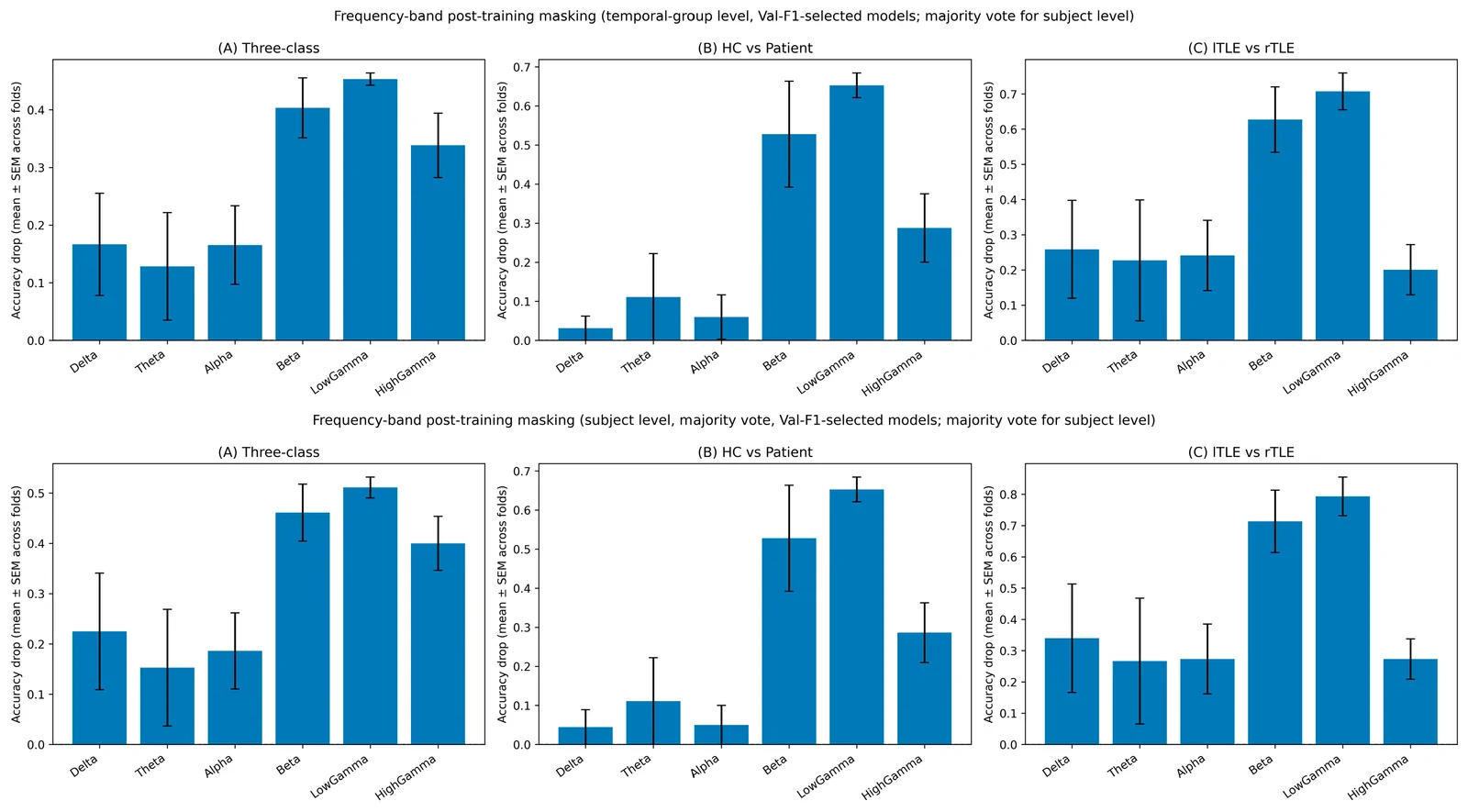
Post-training frequency-band masking analysis (top row: temporal-group level; bottom row: subject level; error bars represent the mean ± SEM across the five folds; frequency bands were masked after training without retraining the model; the HC-vs.-TLE and lTLE-vs.-rTLE analyses were both derived from the predictions of the same three-class model).

To analyze the model’s dependence on information from different frequency bands, a post-training frequency-band masking analysis was performed, as shown in Figure 6. The temporal-group-level and subject-level results showed generally consistent trends. In the three-class classification task, masking the low-gamma band (30–80 Hz) resulted in the largest mean decrease in accuracy, followed by the beta band (13–30 Hz). The high-gamma band (80–250 Hz) also showed relatively high model dependence, whereas the effects of the delta, theta, and alpha bands were comparatively smaller. In the HC vs. TLE and lTLE vs. rTLE analyses further derived from the predictions of the same three-class model, low gamma and beta remained the frequency bands upon which the model showed the greatest dependence. Specifically, in the HC vs. TLE task, the model showed a more pronounced dependence on higher-frequency bands, particularly low gamma. In the lTLE vs. rTLE lateralization task, the relative dependence on lower-frequency bands, including delta, theta, and alpha, increased, whereas the relative ranking of high gamma decreased. Overall, both evaluation levels indicated that low gamma and beta were the frequency bands upon which the model depended most strongly, although the relative contribution patterns of different frequency bands differed to some extent between disease identification and left–right lateralization.

In addition, to further examine the brain regions receiving greater attention from the model within different frequency bands, node-level fusion weights were extracted for analysis. The results are presented in Table 7.

Based on the overall mean fusion weights, different frequency bands exhibited distinct spatial distribution patterns. In the delta band, the highest-ranked regions mainly included HIP.L, PHG.L, HIP.R, and MTG.R. In the theta and alpha bands, regions including STG, PCUN, and SFGmed showed relatively high mean weights. The top 5 regions in the beta band mainly included bilateral ORBsupmed, bilateral DCG, and ORBmid.L. In the low-gamma band, ORBsupmed.R, ORBmid.R, and bilateral DCG ranked among the regions with the highest overall weights. In the high-gamma band, the highest-ranked regions mainly included bilateral IPL, ORBmid.R, ACG.L, and PCUN.R.

The cross-fold top 5 occurrence frequencies showed that the exact regional rankings varied across folds. In the theta band, STG.R and PCUN.R appeared in the top 5 in four of the five cross-validation folds, whereas STG.L and IPL.L appeared in the top 5 in three folds. ORBsupmed.R appeared in the top 5 in three folds in both the beta and low-gamma bands, and IPL.L appeared in the top 5 in three folds in the high-gamma band. As shown in Table 8, the mean pairwise Jaccard index for each of the six frequency bands was higher than the random descriptive reference level. The theta band showed the greatest cross-fold overlap, whereas the alpha band was only slightly above the random reference level. Some regions that ranked among the top 5 based on the overall mean weights across all 43 subjects appeared in the top 5 in relatively few individual cross-validation folds, indicating that the overall mean rankings were not fully consistent with the highest-ranked regions in each fold. Therefore, these results only indicate potentially important brain regions receiving relatively high overall attention from the model within the predefined set of 26 DMN-related ROIs and provide a degree of model interpretability. However, because the exact top 5 rankings varied across folds and the analysis was restricted to predefined ROIs, these regions cannot yet be regarded as stable biological key regions at the whole-brain level.

## 4. Discussion

This study proposes a multiscale directed temporal graph convolutional network (MSD-STGNN) for TLE identification and lateralization classification. In the fivefold cross-validation using subjects as the primary evaluation unit, MSD-STGNN achieved relatively high overall classification performance among the comparison models and ablation configurations investigated in this study, with the highest mean values across all evaluation metrics. Taken together, the comparison and ablation results showed that the complete model achieved relatively high mean classification performance across different connectivity representations, multiband dynamic fusion configurations, and temporal modeling settings, suggesting that jointly modeling connectivity directionality, frequency-band-specific information, and short-term dynamic information may be beneficial for three-class TLE classification.

### 4.1. Role of the Dynamic Multiband Fusion Mechanism

In the subject-level results, the fivefold mean values of all evaluation metrics for both the model without frequency-band fusion and the basic frequency-band fusion model were lower than those of the complete model, suggesting that multiband representations and node-level dynamic frequency-band fusion may be beneficial for three-class TLE classification. Previous studies have shown that TLE-related brain network connectivity and abnormalities in directional information flow exhibit a certain degree of frequency-band specificity, with different frequency bands showing distinct patterns of network alterations [12,13,14]. Therefore, although constructing a single connectivity matrix directly from the full 1–250 Hz frequency range preserves overall connectivity information across a broad frequency range, network features from different frequency bands are integrated into the same representation, which may weaken discriminative differences among frequency bands. This may be one reason for the lower mean classification performance observed in the model without frequency-band fusion.

Compared with the model without frequency-band fusion, the basic frequency-band fusion model weighted different frequency bands using global learnable weights and achieved higher mean values across all subject-level evaluation metrics, suggesting that retaining multiband information may be beneficial for TLE classification. However, the basic frequency-band fusion method learns only a single set of global frequency-band weights and cannot adaptively adjust the contribution of each frequency band according to the feature representations of different subjects, time slices, and brain regions. In TLE, abnormal network patterns may not be confined to a single brain region or a single frequency range, and different brain regions may exhibit different connectivity abnormalities across frequency bands [12,13,14]. Therefore, node-level dynamic frequency-band fusion can adjust the contributions of different frequency bands according to the current feature representation and may be structurally more suitable for characterizing such frequency- and spatially dependent differences. This may be one reason why the complete model achieved better mean classification performance.

After removal of the gated residual correction mechanism, the mean values of the model’s evaluation metrics decreased, suggesting that the gated correction mechanism may have a positive effect on the classification performance of the current model. Structurally, this mechanism adaptively balances the attention-based fusion representation and the mean-fusion representation, allowing the model to retain a certain amount of overall multiband information while utilizing dynamic frequency-band weights, which may reduce excessive dependence on a small number of highly weighted frequency bands. Therefore, the gated residual correction mechanism may be associated with a more reasonable multiband fusion representation.

### 4.2. Differences in Frequency-Band Dependence and Brain-Region Attention Patterns

The frequency-band masking experiments further revealed the model’s dependence on different frequency bands during classification. In the three-class classification task, masking the low-gamma band (30–80 Hz) resulted in the largest decrease in accuracy, followed by the beta band (13–30 Hz), suggesting that the current model exhibited relatively high dependence on information from the low-gamma and beta bands. Previous MEG studies have reported that the mean connectivity strength of patients with TLE is significantly higher than that of healthy controls in the low-gamma band, together with frequency-specific network alterations [12]. Studies of effective connectivity in epilepsy have also suggested that the gamma and beta bands have a certain degree of discriminative value for disease classification [24]. These findings provide some neurophysiological support for the relatively high dependence of the present model on the low-gamma and beta bands.

In the lateralization analysis derived from the predictions of the same three-class model, the model showed increased relative dependence on lower-frequency bands, including delta, theta, and alpha, whereas the relative ranking of high gamma decreased. This suggests that low-frequency information may provide complementary discriminative information for distinguishing left and right TLE. Previous studies have also reported that low-frequency activity has a certain degree of value for TLE lateralization, with the delta band potentially containing discriminative information related to TLE lateralization [11,57]. Abnormal patterns of directional information flow in TLE have also been reported in specific low-frequency bands [14]. It should be noted that the high-gamma band is susceptible to contamination by non-neural artifacts such as electromyographic activity. Although ICA was used during data preprocessing to remove major artifacts, the influence of residual high-frequency artifacts cannot be completely excluded. Therefore, the results related to the high-gamma band should be interpreted with caution.

Node-level fusion weights further indicated that the spatial distribution of model attention differed to some extent across frequency bands. Based on the overall mean weights across the 43 subjects, the highest-ranked regions in the beta band mainly included bilateral ORBsupmed, bilateral DCG, and ORBmid.L, whereas ORBsupmed.R, ORBmid.R, bilateral DCG, and PHG.R showed relatively high mean weights in the low-gamma band. ORBsupmed and ORBmid are regions related to the orbitofrontal cortex, which is involved in reward value representation, emotional processing, and decision-making and participates in behavioral regulation through its connections with regions such as the cingulate cortex [58]. DCG is a cingulate cortex-related region. Previous studies have shown that the cingulate cortex is involved in various cognitive and behavioral processes and may also participate in the propagation of epileptic activity [59,60]. Therefore, the relatively high overall fusion weights of orbitofrontal- and cingulate-related nodes in the beta and low-gamma bands are, to some extent, consistent with previous reports of extra-temporal network abnormalities in TLE [12,22,34,61]. However, these results reflect the degree of model attention to features of the corresponding brain regions within the predefined ROI set and cannot by themselves establish that these regions have specific pathogenic roles. In the high-gamma band, regions with relatively high overall mean weights mainly included bilateral IPL, ORBmid.R, ACG.L, and PCUN.R. ACG is an anterior cingulate-related region, and previous epilepsy studies have suggested that the cingulate cortex may be involved in epileptic network activity and propagation [62]. IPL is an important parietal region involved in various higher-order cognitive processes and network information integration [63], whereas PCUN is an important node of the DMN [38]. Therefore, the relatively high fusion weights of these regions may reflect the model’s use of frontal, cingulate, and parietal network information in this frequency band.

In the lower-frequency bands, the highest-ranked regions in the delta band mainly included medial- and lateral-temporal-lobe-related nodes such as HIP, PHG, and MTG. The theta and alpha bands more frequently involved regions including STG, PCUN, SFGmed, and IPL. HIP and PHG are medial temporal lobe regions that are closely associated with seizure onset and propagation in TLE, as well as related memory dysfunction. Previous studies have also reported altered DMN connectivity in patients with TLE, particularly abnormalities in hippocampal-related connectivity [5,64]. PCUN and SFGmed are important nodes of the DMN, and previous studies have shown that both lTLE and rTLE can be accompanied by changes in DMN connectivity, with some lateralized differences [22,34,64]. Combined with the increased relative dependence on lower-frequency bands observed in the lTLE vs. rTLE analysis in the present study, these findings suggest that the model may utilize low-frequency information from different regions within the predefined DMN to assist lateralization discrimination. This is also consistent with previous MEG studies reporting that low-frequency activity has value for TLE lateralization [57].

In addition to the overall mean weights, the cross-fold reproducibility of regional rankings was further evaluated using the frequency of top 5 occurrences across folds and pairwise Jaccard indices. The results showed that the exact top 5 brain regions varied to some extent across folds, and no region was observed to rank among the top 5 in all five folds. The theta band showed relatively high reproducibility, with STG.R and PCUN.R appearing in the top 5 in four of the five folds. Its mean pairwise Jaccard index was 0.328, higher than the random top 5 reference level of 0.116. By contrast, the Jaccard index for the alpha band was 0.140, only slightly above the random reference level, whereas the values for the remaining frequency bands fell between these two levels. These findings indicate that the important regions reflected by the overall mean weights were not fully consistent with those identified within individual folds, and that the exact regional rankings exhibited a certain degree of cross-fold variability.

Overall, the frequency-band masking and node-level fusion-weight analyses indicated that the model exhibited varying degrees of dependence on information from different frequency bands and brain regions. The model exhibited relatively high dependence on the low-gamma and beta bands, whereas lower-frequency information may provide complementary information for lateralization. Across different frequency bands, regions with relatively high overall fusion weights involved the orbitofrontal, cingulate, medial temporal, and parietal areas. This spatial distribution is, to some extent, consistent with previously reported extra-temporal functional network abnormalities in TLE [12,22,61].

However, it should be emphasized that the node-level interpretation in this study was restricted to the 26 predefined DMN-related brain regions, and the exact rankings of important regions varied across folds. Therefore, the current results are mainly intended to explain the frequency-band and regional information utilized by the model within this predefined ROI set and are more appropriate for describing the model’s overall attention patterns. They are not sufficient to identify stable TLE-related key brain regions or biological biomarkers at the whole-brain level.

### 4.3. Limitations and Future Directions

This study has several limitations. First, the number of independent subjects was limited, and the data were obtained from a single center. Although subject-level fivefold cross-validation was used for internal validation, the generalizability of the model still requires further evaluation in larger cohorts and independent multicenter datasets. Owing to the current sample size, the ablation experiments were mainly used to compare performance changes across different model configurations, and more systematic statistical inference was not performed. In addition, not all available subjects in the rTLE group were included. Although the inclusion procedure was not based on classification results, model performance, or feature information, it may still have introduced some selection bias and reduced the number of independent subjects available for analysis. Moreover, MEG examinations contributed to the original clinical lateralization assessment, while the present model also used MEG-derived brain-network features. Therefore, the current results primarily reflect the model’s internal validation performance relative to the existing clinical lateralization labels. Future studies should prioritize the inclusion of all eligible subjects, preserve the natural class distribution, incorporate more complete and standardized patient-level clinical reference information, and further validate the model in larger independent cohorts.

Second, this study analyzed only 26 predefined DMN-related ROIs. Therefore, the current interpretability results mainly reflect the frequency-band and brain-region attention patterns of the model within this predefined network and cannot be directly generalized to the whole brain. The top 5 brain regions also showed some variability across different cross-validation folds; therefore, the current findings are not sufficient to establish stable TLE-related brain-region biomarkers. Future studies with larger samples and whole-brain ROIs are needed to further evaluate the reproducibility of the observed brain-region and frequency-band patterns.

Third, several methodological considerations related to the DTF construction should be noted. This study uniformly adopted a predefined MVAR order of *p* = 5. Although supplementary analyses indicated relatively high numerical stability and robustness to neighboring model orders, residual diagnostics still suggested some limitations in model adequacy; therefore, *p* = 5 should not be regarded as the uniquely statistically optimal order for all temporal slices. In addition, the interpretation of the 80–250 Hz high-gamma band requires caution. Although this frequency-band definition followed previous multiband MEG analysis schemes in epilepsy, the resampling procedure included anti-aliasing processing, and preprocessing reduced major non-neural artifacts, residual electromyographic activity and other high-frequency non-neural components cannot be completely excluded. Future studies with larger samples should further optimize MVAR order selection and validate the stability and neurophysiological basis of high-frequency connectivity features.

## 5. Conclusions

This study proposes a multiscale directed temporal graph convolutional network (MSD-STGNN) based on MEG-derived directed brain networks for the three-class classification of healthy controls, patients with left temporal lobe epilepsy, and patients with right temporal lobe epilepsy. The model jointly utilizes directional brain-network connectivity, multiband information, and short-term temporal features and showed relatively good classification performance in subject-level fivefold cross-validation. Frequency-band masking and node-level fusion-weight analyses further indicated that the model depended on information from different frequency bands and DMN-related brain regions to varying degrees. In particular, the model exhibited relatively high dependence on the low-gamma and beta bands, while orbitofrontal, cingulate, medial temporal, and parietal regions exhibited relatively high overall fusion weights across different frequency bands. Overall, this study provides a modeling framework with a certain degree of interpretability for the application of MEG-based multiband directed brain networks to automated TLE identification and lateralization classification.

## Figures and Tables

**Table 1:** Demographic and clinical characteristics of the subjects included in this study (all patients with TLE had drug-resistant epilepsy; sMRI showed no evident structural abnormalities in most patients, and no sMRI abnormalities were observed in HCs).

Group	Male/Female	Age (Years)	Disease Duration	Age at First Seizure
HC	6/8	27.90 ± 6.97	—	—
lTLE	9/4	26.08 ± 5.54	7.46 ± 3.23	18.62 ± 6.65
rTLE	11/5	27.75 ± 6.15	10.06 ± 5.59	17.69 ± 7.90

**Table 2:** Selection of DMN Brain Regions.

Anatomical Domain	Anatomical Description	AAL Label	Abbreviation
Orbital Frontal Cortex	Superior frontal gyrus, medial orbital	Frontal_Med_Orb	ORBsupmed
	Middle frontal gyrus, orbital part	Frontal_Mid_Orb	ORBmid
medial prefrontal cortex/anterior cingulate cortex	Superior frontal gyrus, medial	Frontal_Sup_Medial	SFGmed
	Anterior cingulate and paracingulate gyri	Cingulum_Ant	ACG
posterior cingulate/retrosplenial cortex	Median cingulate and paracingulate gyri	Cingulum_Mid	DCG
	Posterior cingulate gyrus	Cingulum_Post	PCG
	Precuneus	Precuneus	PCUN
hippocampus/parahippocampal cortex	Hippocampus	Hippocampus	HIP
	Parahippocampal gyrus	ParaHippocampal	PHG
Inferior parietal lobe	Inferior parietal, but supramarginal and angular gyri	Parietal_Inf	IPL
lateral temporal cortex	Superior temporal gyrus	Temporal_Sup	STG
	Middle temporal gyrus	Temporal_Mid	MTG
	Inferior temporal gyrus	Temporal_Inf	ITG

**Table 3:** Comparison of Results Across Different Models and Ablation Experiments.

Evaluation Level	Method	Accuracy	Precision	Recall	Specificity	F1-Score	AUC
Subject-level	**MSD-STGNN**	**0.836 ± 0.067**	**0.882 ± 0.064**	**0.833 ± 0.056**	**0.913 ± 0.034**	**0.830 ± 0.065**	**0.900 ± 0.055**
	PLI Baseline	0.719 ± 0.070	0.691 ± 0.182	0.700 ± 0.050	0.851 ± 0.035	0.660 ± 0.092	0.823 ± 0.043
	Basic Frequency-Band Fusion	0.722 ± 0.052	0.646 ± 0.128	0.706 ± 0.061	0.859 ± 0.025	0.660 ± 0.095	0.811 ± 0.176
	Without Frequency-Band Fusion	0.675 ± 0.091	0.634 ± 0.135	0.661 ± 0.093	0.833 ± 0.045	0.626 ± 0.105	0.803 ± 0.134
	Without Temporal Modeling	0.697 ± 0.140	0.700 ± 0.183	0.700 ± 0.122	0.842 ± 0.076	0.678 ± 0.134	0.862 ± 0.079
	Without Gated Residual Correction	0.722 ± 0.052	0.700 ± 0.175	0.711 ± 0.099	0.856 ± 0.038	0.663 ± 0.122	0.810 ± 0.082
temporal-group-level	MSD-STGNN	0.778 ± 0.047	0.800 ± 0.033	0.776 ± 0.030	0.886 ± 0.028	0.768 ± 0.036	0.888 ± 0.044
	PLI Baseline	0.659 ± 0.053	0.663 ± 0.026	0.656 ± 0.039	0.827 ± 0.029	0.647 ± 0.033	0.810 ± 0.036
	Basic Frequency-Band Fusion	0.725 ± 0.068	0.733 ± 0.083	0.710 ± 0.066	0.860 ± 0.032	0.695 ± 0.066	0.795 ± 0.163
	Without Frequency-Band Fusion	0.689 ± 0.084	0.671 ± 0.129	0.679 ± 0.084	0.842 ± 0.042	0.651 ± 0.098	0.815 ± 0.121
	Without Temporal Modeling	0.705 ± 0.088	0.747 ± 0.095	0.708 ± 0.075	0.849 ± 0.050	0.687 ± 0.083	0.850 ± 0.055
	Without Gated Residual Correction	0.713 ± 0.039	0.721 ± 0.123	0.702 ± 0.089	0.851 ± 0.034	0.668 ± 0.098	0.812 ± 0.079

**Note:** Bold values indicate the performance of MSD-STGNN at the subject level, which is regarded as the primary evaluation level in this study.

**Table 4:** Class-specific performance at the subject level under fivefold cross-validation.

Class	Precision	Recall	Specificity	F1-Score	AUC
HC	1.000 ± 0.000	0.933 ± 0.149	1.000 ± 0.000	0.960 ± 0.089	0.978 ± 0.050
lTLE	0.853 ± 0.202	0.767 ± 0.224	0.887 ± 0.176	0.777 ± 0.137	0.843 ± 0.126
rTLE	0.793 ± 0.125	0.800 ± 0.298	0.853 ± 0.084	0.754 ± 0.167	0.880 ± 0.084

**Table 5:** Subject-level fivefold cross-validation and pooled OOF performance of MSD-STGNN.

Evaluation	Accuracy	Macro F1	Macro AUC
5-fold CV (mean ± SD)	0.836 ± 0.067	0.830 ± 0.065	0.900 ± 0.055
Pooled OOF (*n* = 43)	0.837	0.840	0.870

**Table 6:** Class-specific performance of pooled OOF predictions at the subject level.

Class	Precision	Sensitivity	Specificity	F1	AUC
HC	1.000	0.929	1.000	0.963	0.938
lTLE	0.769	0.769	0.900	0.769	0.823
rTLE	0.765	0.813	0.852	0.788	0.847

**Table 7:** The fusion weights of the top 5 brain regions in each frequency band and the stability across folds.

Frequency Band	AAL Region ID	AAL Label	Raw Weight	Fold Top 5
Delta	37	HIP.L	0.139579 ± 0.037431	1/5
	39	PHG.L	0.139311 ± 0.037160	0/5
	38	HIP.R	0.138992 ± 0.036724	0/5
	86	MTG.R	0.138990 ± 0.037301	0/5
	36	PCG.R	0.138957 ± 0.036773	0/5
Theta	82	STG.R	0.147411 ± 0.031743	4/5
	81	STG.L	0.147049 ± 0.032097	3/5
	68	PCUN.R	0.146978 ± 0.031990	4/5
	24	SFGmed.R	0.146962 ± 0.032195	2/5
	61	IPL.L	0.146960 ± 0.031784	3/5
Alpha	82	STG.R	0.133445 ± 0.033901	2/5
	81	STG.L	0.133265 ± 0.034020	2/5
	23	SFGmed.L	0.132962 ± 0.033772	2/5
	24	SFGmed.R	0.132893 ± 0.033872	1/5
	85	MTG.L	0.132730 ± 0.033775	1/5
Beta	26	ORBsupmed.R	0.182156 ± 0.047820	3/5
	25	ORBsupmed.L	0.181083 ± 0.048042	3/5
	33	DCG.L	0.179378 ± 0.050442	2/5
	34	DCG.R	0.178817 ± 0.050653	2/5
	9	ORBmid.L	0.177036 ± 0.049705	2/5
Low gamma	26	ORBsupmed.R	0.204362 ± 0.062189	3/5
	10	ORBmid.R	0.204275 ± 0.066110	2/5
	33	DCG.L	0.204027 ± 0.064952	2/5
	34	DCG.R	0.203762 ± 0.065294	2/5
	40	PHG.R	0.203757 ± 0.065831	0/5
High gamma	61	IPL.L	0.206444 ± 0.056939	3/5
	62	IPL.R	0.206333 ± 0.056724	2/5
	10	ORBmid.R	0.205911 ± 0.053844	2/5
	31	ACG.L	0.205611 ± 0.056271	0/5
	68	PCUN.R	0.205564 ± 0.056621	2/5

**Table 8:** Cross-fold Jaccard stability of the top 5 brain-region sets across frequency bands.

Frequency Band	Mean Pairwise Jaccard	Random Top 5 Reference	Difference from Random
Delta	0.219	0.116	+0.103
Theta	0.328	0.116	+0.212
Alpha	0.140	0.116	+0.024
Beta	0.177	0.116	+0.061
Low Gamma	0.157	0.116	+0.041
High Gamma	0.177	0.116	+0.061

## Data Availability

The datasets used and analyzed during the current study are available from the corresponding author upon reasonable request.

## Supplementary Materials

The following supporting information can be downloaded at: https://www.mdpi.com/article/10.3390/s26196185/s1, Supplementary Note S1: Sensitivity Analysis of the MVAR Model to Neighboring Model Orders; Table S1: Consistency of DTF Connectivity Structures Across Neighboring MVAR Model Orders; Supplementary Note S2: Slice-Wise Stability and Residual Diagnostics of the MVAR Model; Table S2: Slice-Wise Stability and Residual Diagnostic Results for the MVAR Model.

## Abbreviations

The following abbreviations are used in this manuscript:

TLE	Temporal Lobe Epilepsy
HCs	Healthy Controls
lTLE	Left Temporal Lobe Epilepsy
rTLE	Right Temporal Lobe Epilepsy
MEG	Magnetoencephalography
DTF	Directed Transfer Function
GNN	Graph Neural Network
AAL	Anatomical Automatic Labeling
DMN	Default Mode Network
ROI	Region of Interest
GRU	Gated Recurrent Unit

## Appendix A. Model Parameters

**Table A1:** Hyperparameter settings of MSD-STGNN.

Parameter	Value	Parameter	Value
Learning rate	5 × 10^−4^	Temperature parameter τ	2.0
Batch size	64	GRU hidden dimension	64
Maximum epochs	80	GRU layers	2
Validation split	20%	GRU dropout	0.3
Early-stopping patience	15	Classifier dimensions	64 → 16 → 3
Directed GCN hidden dimension	24	Classifier dropout	0.5
Attention heads	2	Random seed	42
Attention dropout	0.1	Optimizer	Adam

**Note:** The arrow (→) indicates the dimensional progression of the classifier layers, from a 64-dimensional input to a 16-dimensional hidden layer and a 3-dimensional output.
