# Supplementary material for: Interpretable Multiscale Directed Temporal Graph Learning for MEG-Based Identification and Lateralization of Temporal Lobe Epilepsy
Source: Sensors (Basel). 2026 Sep 29;26(19):6185. doi: 10.3390/s26196185 (PMC13667426; doi:10.3390/s26196185)
Supplement: Supplementary file 1 [file sensors-26-06185-s001.zip › sensors-4486719-supplementary.pdf]

Supplementary Note S1. Sensitivity Analysis of the MVAR Model to Neighboring Model Orders

To assess the stability of DTF connectivity structures under small variations in the MVAR model order around $p = 5$, DTF was additionally recomputed using the neighboring orders $p = 4$and $p = 6$, while all other DTF calculation procedures, frequency-band definitions, and normalization procedures were kept unchanged. The resulting DTF matrices were separately compared with those obtained using $p = 5$in the main analysis.

The consistency of the normalized DTF matrices across different model orders was evaluated using the Spearman correlation coefficient, Pearson correlation coefficient, and relative Frobenius difference. Across the six frequency bands, the Spearman correlation coefficients between $p = 4$ and $p = 5$ ranged from 0.844 to 0.966, the Pearson correlation coefficients ranged from 0.854 to 0.968, and the relative Frobenius differences ranged from 0.143 to 0.421. For $p = 6$versus $p = 5$, the corresponding ranges were 0.868–0.970, 0.880–0.974, and 0.100–0.366, respectively. Overall, the major DTF connectivity patterns remained highly consistent under small variations in model order around $p = 5$, indicating that the downstream DTF representations were robust to changes in neighboring model orders.

Table S1. Consistency of DTF Connectivity Structures Across Neighboring MVAR Model Orders.

Comparison	Spearman Correlation Coefficient	Pearson Correlation Coefficient	Relative Frobenius Difference
$p = 4$ vs $p = 5$	0.844–0.966	0.854–0.968	0.143–0.421
$p = 6$ vs $p = 5$	0.868–0.970	0.880–0.974	0.100–0.366

Supplementary Note S2. Slice-Wise Stability and Residual Diagnostics of the MVAR Model

To further evaluate the numerical stability of the $p = 5$MVAR model used in the main analysis, slice-wise stability was assessed across all 1,720 temporal slices from the 43 subjects. The spectral radius of the MVAR companion matrix was used as the stability criterion, and the model was considered to satisfy the stability condition when the spectral radius was $\rho < 1$.

Among all 1,720 temporal slices, 1,719 satisfied $\rho < 1$, corresponding to an overall stability rate of 99.94%. Only one temporal slice marginally exceeded the stability boundary, with a spectral radius of $\rho = 1.000971$. Therefore, the $p = 5$MVAR model used in the main analysis exhibited nearly complete numerical stability in the current dataset.

In addition, the residual diagnostic procedure implemented in ARfit was used to further evaluate how well the $p = 5$model accounted for the temporal dependence in the original time series. The residual diagnostics were performed using a maximum lag of 20 samples and a significance level of $\alpha = 0.05$. Among the temporal slices for which valid residual diagnostic results were obtained, the mean absolute autocorrelation decreased from 0.212 in the raw time series to 0.136 in the residuals, with a mean raw-to-residual ACF reduction of 31.51%. This indicates that MVAR fitting accounted for part of the temporal dependence present in the original time series.

At the same time, the stringent modified Li–McLeod test for multivariate residual uncorrelatedness was not satisfied. Therefore, $p = 5$was not interpreted in this study as providing a fully adequate fit for all temporal slices or as the uniquely statistically optimal model order. Instead, it was treated as a predefined and uniform parameter that exhibited high

numerical stability and whose downstream DTF structures showed good robustness to variations in neighboring model orders.

Table S2. Slice-Wise Stability and Residual Diagnostic Results for the MVAR Model.

Metric	Result
Stable temporal segments	1719/1720
Stability rate	99.94%
Marginally unstable segment	$\rho = 1.000971$
Raw mean absolute ACF	0.212
Residual mean absolute ACF	0.136
Mean raw-to-residual ACF reduction	31.51%
